# Constructing Dynamic Functional Networks via Weighted Regularization and Tensor Low-Rank Approximation for Early Mild Cognitive Impairment Classification

**DOI:** 10.3389/fcell.2020.610569

**Published:** 2021-01-11

**Authors:** Zhuqing Jiao, Yixin Ji, Jiahao Zhang, Haifeng Shi, Chuang Wang

**Affiliations:** ^1^School of Microelectronics and Control Engineering, Changzhou University, Changzhou, China; ^2^School of Computer Science and Artificial Intelligence, Changzhou University, Changzhou, China; ^3^Department of Radiology, Changzhou Second People’s Hospital Affiliated to Nanjing Medical University, Changzhou, China; ^4^School of Medicine, Ningbo University, Ningbo, China

**Keywords:** dynamic functional network (DFN), weighted regularization (WR), tensor low-rank approximation (TLA), early mild cognitive impairment (eMCI), classification

## Abstract

Brain functional networks constructed via regularization has been widely used in early mild cognitive impairment (eMCI) classification. However, few methods can properly reflect the similarities and differences of functional connections among different people. Most methods ignore some topological attributes, such as connection strength, which may delete strong functional connections in brain functional networks. To overcome these limitations, we propose a novel method to construct dynamic functional networks (DFN) based on weighted regularization (WR) and tensor low-rank approximation (TLA), and apply it to identify eMCI subjects from normal subjects. First, we introduce the WR term into the DFN construction and obtain WR-based DFNs (WRDFN). Then, we combine the WRDFNs of all subjects into a third-order tensor for TLA processing, and obtain the DFN based on WR and TLA (WRTDFN) of each subject in the tensor. We calculate the weighted-graph local clustering coefficient of each region in each WRTDFN as the effective feature, and use the *t*-test for feature selection. Finally, we train a linear support vector machine (SVM) classifier to classify the WRTDFNs of all subjects. Experimental results demonstrate that the proposed method can obtain DFNs with the scale-free property, and that the classification accuracy (ACC), the sensitivity (SEN), the specificity (SPE), and the area under curve (AUC) reach 87.0662% ± 0.3202%, 83.4363% ± 0.5076%, 90.6961% ± 0.3250% and 0.9431 ± 0.0023, respectively. We also achieve the best classification results compared with other comparable methods. This work can effectively improve the classification performance of DFNs constructed by existing methods for eMCI and has certain reference value for the early diagnosis of Alzheimer’s disease (AD).

## Introduction

Alzheimer’s disease (AD) is a typical dementia disease, which accounts for about 60–70% of patients with dementia diseases (Atangana et al., 2018). Notably, AD is a neurodegenerative disease mainly characterized by memory dysfunction and with an increasing morbidity, mortality, and economic cost (Lu et al., 2019). However, it is unclear that AD biomarkers are critical for catching the disease early to allow for preventative interventions. Mild cognitive impairment (MCI) is a transitional state between normal senility and AD (Muldoon and Bassett, 2016). Recent researches show that about 10–12% of MCI patients deteriorate to AD patients every year, while only 1–2% of normal senilities deteriorate to AD patients every year. MCI patients are considered to be a high-risk group among AD patients (Jiao et al., 2014; Zhang et al., 2015b). The brains of patients with early mild cognitive impairment (eMCI) have very subtle changes compared with those of normal people, which mainly manifest in abnormal functional connections between certain regions (Bi et al., 2020a,b; Tobia et al., 2017). If treatment and intervention can be carried out in time after eMCI is discovered, we can greatly delay or prevent the development of eMCI to MCI and AD.

Interestingly, non-genetic AD or MCI biomarkers are also being extensively explored. With the development of neuroimaging techniques, data collection technologies and analysis methods are constantly being improved. Scientists can directly and non-invasively study the structure and functional activities of brains, thereby they can reveal the functional and structural characteristics from the brains. Neuroimaging techniques have become important tools for humans to explore and study the brains (Zhang and Raichle, 2010; Zhang et al., 2016b). Specially, the spatial resolution of functional magnetic resonance imaging (fMRI) technology can reach the millimeter level, and fMRI has the characteristics of higher temporal resolution at the same time. Thus, it has been widely used in clinical and scientific research. Compared with task-state fMRI, resting-state fMRI has advantages such as simple and easy operation, which provides great convenience for the diagnosis of brain diseases (Du et al., 2012; Zhang et al., 2015a). However, the spontaneous brain activity and the state of the scanning instruments are usually random and asynchronous, so it is a big challenge to identify eMCI patients with resting-state fMRI (Wee et al., 2016a). Brain functional networks can effectively describe the way of transmitting information inside the brain, especially based on resting-state fMRI, have been widely applied to the study of brain diseases (Zhang et al., 2018a,b; Bi et al., 2021).

Currently, *Pearson*’s correlation (PC) is one of the most common methods to construct brain functional networks. However, brain functional networks constructed by PC are often dense and with a large number of redundant and false functional connections (Lee et al., 2011). To solve this problem, one solution is to set some thresholds (Jiao et al., 2018); another solution is to use partial correlation method to construct sparse brain functional networks (Marrelec et al., 2006; Peng et al., 2012; Zhou et al., 2018). In fact, both methods have drawbacks. There is no uniform requirement for the setting of threshold in the method of thresholding processing, while usually a singular solution is obtained by the partial correlation method which involves the problem of solving the inverse of the matrix. The regularization method converts some prior information into regularization terms, which not only fits the data well but also effectively utilizes the prior information. It makes the method for constructing networks scalable (Karen et al., 2018).

The regularization method based on *L*_1_-norm has been successful in the construction of brain functional networks and in the auxiliary diagnosis of brain diseases. For example, sparse representation (SR) (Lee et al., 2011) is a regularization method based on *L*_1_-norm to construct brain functional networks (Liu et al., 2010). But, it has many deficiencies. First, due to the same penalty constraints, there is a lot of noise in constructed networks, which may lose some important functional connections (Li W.K. et al., 2020). Second, this method does not consider the relationships among different subjects, and sparsity constraint, when applied at an individual level, will cause inter-subject variability and reduce classification performance (Wee et al., 2016b). Wee et al. (2014) used group-constrained sparse representation (GSR) to overcome these limitations, and introduced the *L*_2_,_1_-norm regularization term into the construction model of brain functional networks. GSR makes brain functional networks of different people share the similar topological structure, but the group-constraint cannot well reflect the differences of functional connections among different people.

The data in the brain functional network is high-dimensional and redundant, and most of the high-dimensional data is located in a low-dimensional subspace (Meinshausen and Bühlmann, 2006). Relevant studies have shown that high-dimensional redundant data can be obtained by calculating the low-rank approximation of the matrix, so matrix low-rank approximation (MLA) is a solution to find useful information from complex data (Halko et al., 2010). Compared with MLA, tensor low-rank approximation (TLA) can fully consider the correlation and prior knowledge between the data, remove the redundant information of the data, and retain valuable information (Jiang et al., 2019). Jiang et al. (2019) used TLA to make the brain functional networks have similar but not necessarily identical topology across subjects. Although TLA can effectively overcome the limitations of GSR, it is difficult to well reflect some discriminative functional connections. This is a very common disadvantage, because some prior knowledge may be explained by discriminative topology properties in brain functional networks, and the classification performance may be determined by some discriminative functional connections.

To address above problems, we propose a method for constructing dynamic functional networks (DFN) via weighted regularization (WR) and TLA (WRTLA), and apply it to distinguish eMCI subjects from normal subjects. First, we formulate PC as an optimization model, and integrate the connection strength into WR and derive the DFN based on WR (WRDFN). Then we combine all the WRDFNs of all subjects into a third-order tensor for TLA processing and obtain the DFN based on WR and TLA (WRTDFN) of each subject in the tensor. Next, we calculate the weighted-graph local clustering coefficient of each brain region as an effective feature and use *t*-test to select features from WRTDFNs. Then, we train a linear support vector machine (SVM) to classify the WRTDFNs of all subjects and evaluate the classification results. Finally, we discuss the classification performance of WRTDFNs with different regularization parameters, the influence of window widths and step sizes, and discriminative regions and functional connections.

## Materials and Methods

### Framework

**FIGURE 1:**
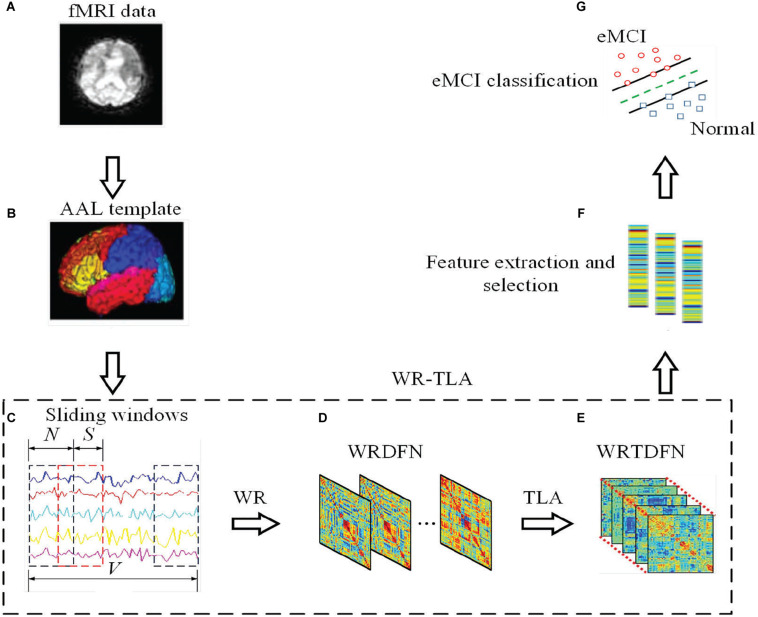
The framework of constructing DFN via WRTLA for eMCI classification, including the following steps: **(A)** Preprocessing the original resting fMRI data of normal subjects and eMCI subjects, **(B)** Extracting the time series containing all brain regions according to the AAL template, **(C)** Using sliding window method to divide the entire time series into several overlapping segments, **(D)** Introducing the WR term into the construction of DFNs to obtain WRDFNs, **(E)** Stacking all WRDFNs of subjects into a tensor and optimizing it using TLA and obtaining WRTDFNs, **(F)** Calculating and extracting the weighted-graph local clustering coefficient of each brain region in WRTDFNs as the effective feature, and using the *t*-test to select features, and **(G)** Training a linear SVM classifier to classify the WRTDFNs of all subjects and evaluating the classification performance.

Figure 1 shows the framework of constructing DFNs via WRTLA for eMCI classification.

### Data Acquisition and Processing

The resting-state fMRI data is derived from the ADNIGo and ADNI2 datasets^1^ of the Alzheimer’s disease neuroimaging initiative (ADNI) project. A total of 60 subjects are selected, including 30 normal subjects and 30 eMCI subjects. The raw resting-state fMRI data is preprocessed by using the SPM8 toolbox^2^ of Matlab R2012a software with further correction and normalization. The process of preprocessed operations contains slice timing, realignment, spatial normalization, smoothing, detrend, filtering, etc. Then, we use the Anatomical Automatic Labeling (AAL) template in the DPARSF^3^ toolbox to divide the brain into 90 brain regions (45 brain regions for the left and right brains), and the time series of each brain region are extracted (Tzourio-Mazoyer et al., 2002). The filtering range is 0.01–0.08 Hz, the standardized bounding box is [−90, −126, −72; 90, 90, 108], and the voxel size is [3 3 3]. It takes a certain amount of time for both the machine and the subjects to enter a stable state. The first 3 time points are removed during preprocessing, and the remaining 137 time points are used for subsequent analysis. Subjects’ data with large head movements (translation > 2 mm, rotation > 2°) are removed after realigning.

### Conventional DFN Construction

Conventional DFNs are constructed by using sliding windows (Chen et al., 2016, 2017). Suppose *X* = [_x1_,_x2_,…,_x*p*_] ∈ ^ℝ*V*×*P*^ is a time series matrix, *V* is the length of the time series, *P* is the number of brain regions, and _x*i*_ ∈ ^ℝ*V*×1^ are the time series of the *i*-th brain region. Assuming that the window width is *N* and the step size is *S*, the total number of windows *K* is expressed as $K=\frac{V-N}{S}+1$. Then, we calculate the PC coefficient between time series in each sliding window. $x_{i}^{k}\in {\mathbb{R}}^{N},\left(k=1,\ldots ,K\right)$ denotes the time series of the *i*-th brain region in the *k*-th window, and the time series matrix $X^{\left(k\right)}=\left[x_{1}^{k},x_{2}^{k},\ldots ,x_{P}^{k}\right]\in {\mathbb{R}}^{P\times N}$ in the *k*-th window concatenate $x_{i}^{k}$ in series. After centralizing and normalizing, the correlation coefficient matrix of brain functional network ***W***^(^*^*k*^*^)^ in the *k*-th sliding window is constructed as follows:

(1) $$W^{\left(k\right)}={\left(X^{\left(k\right)}\right)}^{T}X^{\left(k\right)}$$

### DFN Construction Based on WR

Li et al. (2017) reconstructed the PC method as an optimization model and introduced WR into the construction of the bbrain functional networks, while Yu et al. (2017) adopted weighted sparse representation (WSR) to construct brain functional networks. We refer the former method, which formulate PC into an optimized model and add a WR term based on connection strength. The WR term is used to restrict the strength of each connection in brain functional networks. On this basis, the objective function can be formulated as:

(2) minW∑i=1K∥W(k)-X(k)TX(k)∥F2+λ⁢∑i,j=1nci⁢j|wi⁢j(k)|

where ${∥\cdot ∥}_{F}^{2}$ represents the square of the *F*-norm, and ***X***^(^*^*k*^*^)^ represents the time series of the *k*-th window. λ is a regularization parameter that controls the degree of sparsity, and $w_{i\,j}^{\left(k\right)}$ represents the correlation coefficient between the time series of the *i*-th region and the time series of the *j*-th region in the *k*-th window. *c*_*ij*_ represents the weight penalty for the functional connection between the *i*-th region and the *j*-th region, and we define *c*_*ij*_ as follows:

(3) ci⁢j=exp⁡(-wi⁢j(k)2σ)

where σ is a parameter used to adjust the decay speed of the weight of the corresponding connection strength. The solution method is to calculate the standard deviation (STD) of the absolute value of all elements in the correlation coefficient matrix of brain functional networks.

In Eq. (2), the fitting term is derivable, but the regularization term based on *L*_1_-norm is a convex function and is non-differentiable. To solve the non-differentiable objective function, we use the proximal operator method (Yan et al., 2013) to optimize and solve the objective function. First, we calculate the derivative of Eq. (2):

(4) ∇W(k)f⁢(X(k),W(k))=2⁢(W(k)-X(k)TX(k))

The proximal operator based on *L*_1_-norm is obtained with the expression as follows:

$${p\,r\,o\,x\,i\,m\,a\,l}_{{\lambda ∥.∥}_{1}}\left(W^{\left(k\right)}\right)$$

(5) $$={\left[s\,g\,n\,\left(w_{i\,j}\right)\times m\,a\,x\,\left(a\,b\,s\,\left(w_{i\,j}\right)-\lambda \,c_{i\,j}\right),0\right]}_{N\times N}$$

where ∥⋅∥_1_ represents the *L*_1_-norm. After each gradient descent calculation is completed, the proximal operator can be used to solve the constraint of ***W***^(^*^*k*^*^)^.

The correlation coefficient matrices of the WRDFNs constructed by regularization methods are mostly asymmetric without constraints. Therefore, we adopt the same strategy in the study as Elhamifa and Vidal (2013) to perform symmetry operation on ***W***^(^*^*k*^*^)^ and obtain W*=W(k)+W(k)T2 to represent the correlation coefficient matrix of WRDFN.

### Tensor Low-Rank Approximation

Jiang et al. (2019) adopted TLA to optimize the tensor composed of brain functional networks of different subjects via SR. The specific method is to “assemble” all brain functional networks of different subjects together to form a third-order tensor and then use tensor robust principal component analysis (TRPCA) to optimize the third-order tensor (Lu et al., 2016). Separately, we stack the WRDFNs of all subjects into a third-order tensor and then use TLA to process this tensor from which the WRTDFN of each subject is obtained. The optimization problems in TLA are as follows:

(6) $$\min_{\Omega ,E}{∥\Omega ∥}_{*}+\gamma \,{∥E∥}_{1}\text{ s}.\text{t}.W=\Omega +E$$

where Ω ∈ ^ℝ*N*×*N*×*K*^ is the low-rank tensor finally obtained, and _∥⋅∥*_ represents the trace norm. *E* ∈ ^ℝ*N*×*N*×*K*^ is the noise tensor representing the difference between *W* ∈ ^ℝ*N*×*N*×*K*^ and Ω.***W*** is combined by WRDFNs of all subjects, and γ is a percentage parameter, which is used to remove some weak connections and can be calculated by $1/\sqrt{N\times K}$.

Since the objective function in Eq. (2) is a convex function, we adopted the alternating direction method of multipliers (ADMM) algorithm to solve the objective function (Zhang et al., 2014). The augmented Lagrange multiplier is defined as follows:

$$L_{\mu }\left(\Omega ,E,Y\right)={∥\Omega ∥}_{*}+Y^{T}\left(\Omega +E-W\right)$$

(7) $$+\frac{\mu }{2}\,{∥\Omega +E_{k}-W∥}_{F}^{2}$$

where ***Y*** is the Lagrange multiplier matrix, and μ is the iterative step size of the dual variable in the augmented Lagrange function.

According to the solution framework of the ADMM algorithm, each tensor and variable can be solved and updated as follows:

(8) $$\Omega_{k+1}=\underset{\Omega }{\arg\min}\left({∥\Omega ∥}_{*}+\frac{\mu_{k}}{2}\,{∥\Omega +E_{k}-W+\frac{Y_{k}}{\mu_{k}}∥}_{F}^{2}\right)$$

(9) $$E_{k+1}=\underset{\Omega }{\arg\min}\left(\gamma \,{∥E∥}_{1}+\frac{\mu_{k}}{2}\,{∥\Omega_{k+1}+E-W+\frac{Y_{k}}{\mu_{k}}∥}_{F}^{2}\right)$$

(10) $$Y_{k+1}=Y_{k}+\mu_{k}\left(\Omega_{k+1}+E_{k+1}-W\right)$$

(11) $$\mu_{k+1}=\min\left(\rho \,\mu_{k},\mu_{\max}\right)$$

where ***Y****_*k*_* is the dual matrix in the *k*-th iteration, μ*_*k*_* is the iteration step size of the *k*-th dual variable, and ρ is the ratio of increasing μ in each iteration.

The convergence conditions of the ADMM algorithm are:

$${∥\Omega_{k+1}-\Omega_{k}∥}_{\infty }\leq 10^{-8},{∥E_{k+1}-E_{k}∥}_{\infty }\leq 10^{-8},$$

(12) $${∥\Omega_{k+1}+E_{k+1}-W∥}_{\infty }\leq 10^{-8}$$

### Feature Extraction, Feature Selection, and Classification

The weighted-graph local clustering coefficient describes the local connectivity of the network and quantifies the density of the local structure, which generally decreases with increasing node degree (Jiao et al., 2019). Assuming that there is a network of *N* nodes, the weighted-graph local clustering coefficient of node *i* is defined as:

(13) $$C_{i}=\frac{2\,\sum_{i,j\in v_{i}}{\left(\omega_{i\,j}\right)}^{\frac{1}{3}}}{\left|v_{i}\right|\,\left(\left|v_{i}\right|-1\right)}$$

where _ω*ij*_(*i* ≠ *j*) represents the weight of the connection between node *i* and node *j*, ***v****_*i*_* denotes the set of nodes directly connected to node *i*, and | ***v****_*i*_*| represents the number of elements in ***v****_*i*_*.

SVM has unique advantages in solving small samples, non-linear and high-dimensional data (Li et al., 2018). The goal of SVM is to find an optimal hyperplane that maximizes the separation between each type of sample and the hyperplane, so as to classify the samples. In this study, we extract the weighted-graph local clustering coefficients in WRTDFN as features, use the simplest *t*-test for feature selection, and finally perform the linear SVM classifier (*C* = 1) to classify the WRTDFNs of different subjects. It should be noted that only the training dataset participates in the feature selection process. The dimension of the test dataset is reduced according to the feature index of the training dataset after feature selection, and then the test dataset is classified by the trained classifier. To characterize the classification performance, we use four metrics including classification accuracy (ACC), sensitivity (SEN), specificity (SPE), and area under curve (AUC) (Li et al., 2019). Let TP, TN, FP, and FN denote true positive, true negative, false positive, and false negative, respectively. Then, ACC, SEN, and SPE can be respectively defined as:

(14) $$\text{ACC}=\frac{\mathrm{TP}+\mathrm{TN}}{\mathrm{TP}+\mathrm{TN}+\mathrm{FP}+\mathrm{FN}}$$

(15) $$\text{SEN}=\frac{\text{TP}}{\mathrm{TP}+\mathrm{FN}}$$

(16) $$\text{SPE}=\frac{\text{TN}}{\mathrm{TN}+\mathrm{FP}}$$

## Results

### Visualization of Brain Functional Networks

**FIGURE 2:**
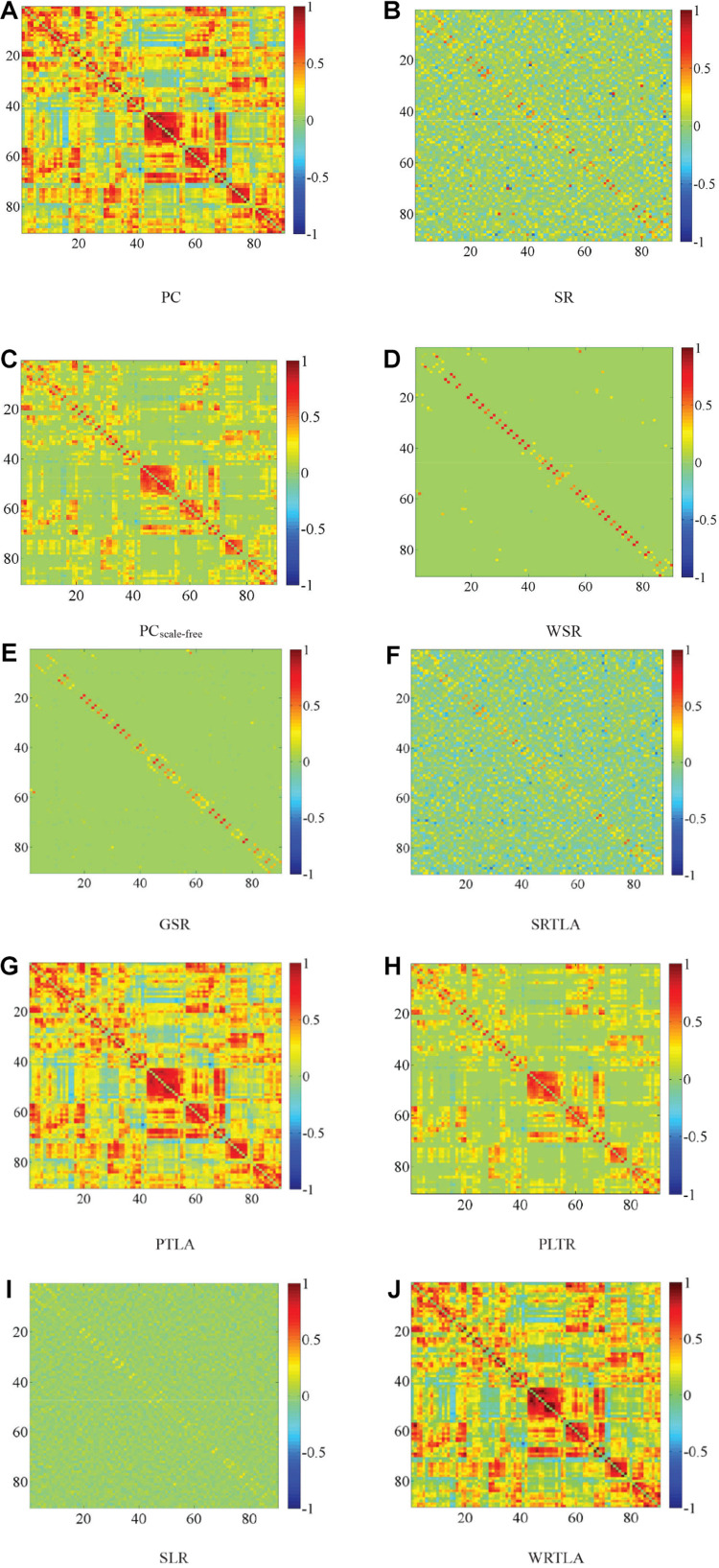
Visualized results of brain functional networks in the same time window. The brain functional network in **(A)** is dense, whereas the brain functional network in **(B)** is sparse, and there is a lot of noise in it. The topologies of brain functional networks in **(F,G)** are clearer than those in **(A,B)** respectively, which indicate that TLA has not changed topologies much but effectively removed some noise connections to improve qualities of brain functional networks, which have certain modularity. The brain functional network in **(E)** is sparser than that in **(F)**, and the brain functional network in **(H)** and **(I)** is sparser and more modular than those in **(A,B)** respectively. In **(D,J)**, some strong functional connections are enhanced, while some weak functional connections are suppressed, which reflects the effectiveness of introducing the weight penalty regularization term. In addition, as shown in **(C)**, we obtain a clearer brain functional network with Hub structure because of the WR term, but some strong functional connections are also penalized.

Zhang and Wang (2015c) discussed the influence of window width and step size in the classification of eMCI patients. They found that the classification results were best when the window width was set to 70 and the step size was set to 1. The DFNs constructed by their methods have many parameters, as well as a large number of samples result from sliding windows. Like WRTLA, it is difficult to use nested cross-validation to select optimal parameters. Setting the same window width and step size facilitates the comparison of classification results with different methods, as shown in Figure 2. The comparable methods include PC and SR (Jiang et al., 2019), the PC_scale–free_ method based on PC and scale-free prior proposed by Li et al. (2017), the WSR proposed by Yu et al. (2017), the group-constrained sparse representation (GSR) proposed by Wee et al. (2014), the TLA method based on sparse representation (SRTLA) and the TLA method based on PC (PTLA) proposed by Jiang et al. (2019), the low-rank tensor regularization method based on PC (PLTR) involved in the study by Gao et al. (2020), and the sparse low-rank representation method (SLR) based on partial correlation proposed by Qiao et al. (2016). Table 1 shows the data-fitting terms and the regularization terms in some aforementioned methods. It is worth noting that all brain functional networks constructed by these methods are DFNs. We select a subject randomly and visualize the brain functional network in the same time window, as shown in Figure 2.

**TABLE 1:** Data-fitting terms and regularization terms in some aforementioned methods.

**Method**	**Data-fitting term**	**Regularization term**
PC (Jiang et al., 2019)	${∥W-X^{T}X∥}_{F}^{2}$	N/A
SR (Jiang et al., 2019)	${∥X-X\,W∥}_{F}^{2}$	λ∥*W*∥_1_
PC_scale–free_ (Li et al., 2017)	${∥W-X^{T}X∥}_{F}^{2}$	$\sum_{i,j=1}^{n}\gamma_{i\,j}\left|w_{i\,j}\right|$
WSR (Yu et al., 2017)	${∥X-X\,W∥}_{F}^{2}$	λ∥*C*⊙*W*∥_1_
GSR (Wee et al., 2014)	${∥X-X\,W∥}_{F}^{2}$	λ_∥*W*∥2,1_
PLTR (Gao et al., 2020)	${∥W-X^{T}X∥}_{F}^{2}$	_λ1_∥*W*∥_1_ + _λ2__∥*W*∥*_
SLR (Qiao et al., 2016)	${∥X-X\,W∥}_{F}^{2}$	_λ1_∥*W*∥_1_ + _λ2__∥*W*∥*_

Since the Hub structure does not have a unified metric, we refer to the method of Jiang et al. (2019) and design the following steps. Firstly, we find the top ten nodes with the largest degree in the brain functional network and calculate the sum of these ten node degrees. Secondly, we divide the sum of these ten node degrees by the sum of all node degrees to get a percentage value representing the Hub score. The higher the Hub score, the more obvious the Hub structure. We calculate the Hub score of the brain functional network via WRTLA with that via PC_scale–free_, and find the former is 15.16% and higher than the latter which is 14.88%. The brain functional network constructed by PC_scale–free_ has an obvious scale-free characteristic.

### Classification Results

We calculate the weighted-graph local clustering coefficient of each region as the effective feature, and use *t*-test to select the feature, with the significance level of 0.05. Then, we employ a SVM with a linear kernel to classify all subjects, which is implemented by using LIBSVM toolbox (Chang and Lin, 2011). The eMCI subjects are treated as positive samples and normal subjects as negative samples. We use ACC, SEN, SPE, and AUC at the end of each classification to evaluate the effectiveness of different methods, and verify the classification results by 10-fold cross-validation (Friston et al., 2003). Specifically, all features are divided into 10 parts, of which 1 part is left for testing in each cross-validation, and the remaining 9 parts are used for training. The regularization parameter is λ = 0.2. The process is repeated 10 times independently, and the average value of each classification after 10 times of 10-fold cross-validation is taken as the final result. Table 2 shows the classification results of different methods with their STD, where the highlighted results indicate the best classification performance.

**TABLE 2:** Classification results of different methods.

**Method**	**ACC (%) ± STD**	**SEN (%) ± STD**	**SPE (%) ± STD**	**AUC ± STD**
SRTLA (Jiang et al., 2019)	47.9853 ± 0.2863	51.7647 ± 8.1956	44.2059 ± 8.1071	0.5530 ± 0.0120
SLR (Qiao et al., 2016)	48.7990 ± 0.3749	50.5833 ± 10.7991	47.0147 ± 10.7029	0.6210 ± 0.0149
SR (Jiang et al., 2019)	54.9559 ± 0.4476	65.7353 ± 1.4451	44.1765 ± 1.8784	0.5665 ± 0.0038
GSR (Wee et al., 2014)	65.4730 ± 0.0722	64.7206 ± 0.1267	66.2255 ± 0.1498	0.7006 ± 0.0003
WSR (Yu et al., 2017)	73.4975 ± 0.3749	73.5343 ± 0.3758	73.4608 ± 0.5484	0.8125 ± 0.0019
PC (Jiang et al., 2019)	85.7070 ± 0.2840	82.5980 ± 0.3365	89.8529 ± 0.2806	0.9373 ± 0.0019
PLTR (Gao et al., 2020)	85.7672 ± 0.2064	83.0931 ± 0.3842	88.4412 ± 0.3167	0.9176 ± 0.0009
PTLA (Jiang et al., 2019)	86.7623 ± 0.1684	82.9069 ± 0.4108	90.6176 ± 0.1964	0.9410 ± 0.0011
PC_scale–free_ (Li et al., 2017)	86.7034 ± 0.3064	83.4951 ± 0.5754	89.9118 ± 0.3526	0.9413 ± 0.0025
WRTLA	87.0662 ± 0.3202	83.4363 ± 0.5076	90.6961 ± 0.3250	0.9431 ± 0.0023

As shown in Table 2, the classification performance of WRTLA is better than other methods. In particular, its ACC, SEN, SPE, and AUC are 87.0662% ± 0.3202%, 83.4363% ± 0.5076%, 90.6961% ± 0.3250% and 0.9431 ± 0.0023, respectively. PC_scale–free_ achieves the classification performance second only to WRTLA, and its ACC, SEN, SPE, and AUC are 86.7034% ± 0.3064%, 83.4951% ± 0.5754%, 89.9118% ± 0.3526% and 0.9413 ± 0.0025, respectively. In addition, the ACC of PLTA is higher than that of PC method, but the ACC of SRTLA is not as high as that of SR. It means that TLA can effectively remove noise connections to improve the quality of DFNs and improve the ACC, but it is not ideal for the SR. The classification performance of WSR and GSR are better than that of SR, which shows that the WR term and the *L*_2_,_1_-norm regularization term are conducive to constructing a more biologically meaningful DFN, and can improve the classification performance of brain diseases. In addition, WRTLA has a higher ACC compared with PLTR and SLR. WRTLA, PLTR and SLR all introduce modularity priors. The classification accuracy of WRTLA and PLTR are higher than PC, but the classification accuracy of SLR is not as good as SR. It indicates that the introduction of modularity priors in the construction of DFN based on PC is beneficial to improve the classification performance, but the introduction of modular priors in the construction of DFN based on SR may not improve classification performance well.

**FIGURE 3:**
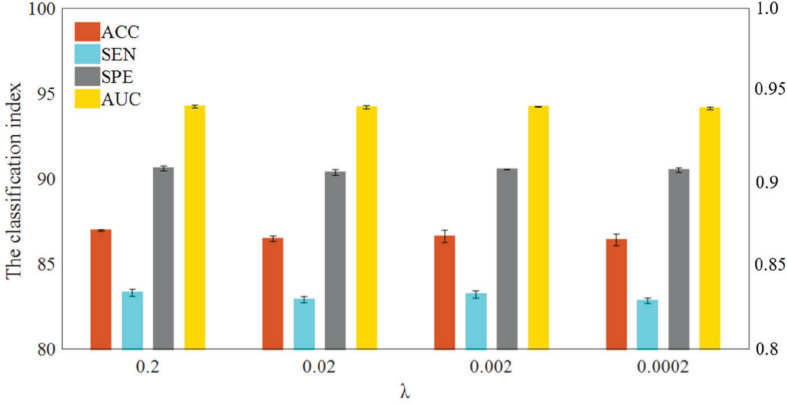
Classification performance of WRTLA with different regularization parameters.

In WRTLA, the purpose of *L*_1_-norm regularization term is mainly to remove redundant features and improve generalization performance of the model. The regularization parameter λ is used to adjust the complexity of the model. We test the classification performance between normal subjects and eMCI subjects with different regularization parameters. Figure 3 shows the specific results, where the range of λ is [2^–4^, 2^–3^, 2^–2^, 2^–1^] (Li et al., 2019; Xu et al., 2020). As can be seen, the classification performance iterating through four parameters just changes a little, but it still has differences. Specifically, the best classification performance is achieved when λ = 0.2, and ACC, SEN, SPE, and AUC are 87.0662% ± 0.3202%, 83.4363% ± 0.5076%, 90.6961% ± 0.3250%, 0.9431 ± 0.0023, respectively. With the changes of λ, the range of the classification performance is not very obvious but shows a downward trend. The reason for this situation may be: as the λ decreases, the features that are useful for classification are also less and less likely to be selected, which ultimately leads to the fitting ability of the classification model worse.

Since the number of samples is very big, all subjects are repeatedly classified to find the optimal combination of window width and step size. Table 3 shows the classification results by different combinations of window widths and step sizes, where the highlighted results indicate the best classification performance. Among them, the step sizes vary from 1 to 4, the window widths vary from 50 to 80, and the regularization parameter *λ* = 0.2.

**TABLE 3:** Classification results by different combinations of window widths and step sizes.

**Method**	**ACC (%) ± STD**	**SEN (%) ± STD**	**SPE (%) ± STD**	**AUC ± STD**
*S* = 1,*V* = 50	83.3598 ± 0.3866	78.3068 ± 0.6416	88.4129 ± 0.4572	0.9153 ± 0.0032
*S* = 2,*V* = 50	88.2852 ± 0.2457	88.7704 ± 0.4030	87.8000 ± 0.2881	0.9409 ± 0.0009
*S* = 3,*V* = 50	80.2386 ± 0.2732	71.5303 ± 0.4681	88.9470 ± 0.3183	0.8822 ± 0.0025
*S* = 4,*V* = 50	79.7609 ± 0.6082	78.9855 ± 0.7358	80.5362 ± 1.1715	0.8981 ± 0.0036
*S* = 1,*V* = 60	84.9487 ± 0.3173	80.4060 ± 0.5459	89.4915 ± 0.2446	0.9288 ± 0.0019
*S* = 2,*V* = 60	90.0208 ± 0.3655	88.5250 ± 0.6074	91.5167 ± 0.2570	0.9504 ± 0.0007
*S* = 3,*V* = 60	87.8025 ± 0.1845	89.8272 ± 0.5466	85.7778 ± 0.5033	0.9462 ± 0.0016
*S* = 4,*V* = 60	81.3750 ± 0.4552	81.2333 ± 0.6992	81.5767 ± 0.7305	0.9132 ± 0.0020
*S* = 1,*V* = 70	86.9975 ± 0.2910	83.3235 ± 0.5850	90.6716 ± 0.3126	0.9429 ± 0.0020
*S* = 2,*V* = 70	87.0797 ± 0.6694	85.6377 ± 1.4025	88.5217 ± 0.8078	0.9351 ± 0.0034
*S* = 3,*V* = 70	87.2464 ± 0.6832	86.1304 ± 0.9831	88.3623 ± 0.8987	0.9343 ± 0.0027
*S* = 4,*V* = 70	83.9259 ± 0.7332	82.1481 ± 1.2103	85.7037 ± 0.4434	0.9204 ± 0.0023
*S* = 1,*V* = 80	87.6782 ± 0.2204	84.7184 ± 0.3256	90.6379 ± 0.3599	0.9448 ± 0.0015
*S* = 2,*V* = 80	87.3222 ± 0.7346	85.1889 ± 1.1536	89.4556 ± 0.6141	0.9310 ± 0.0035
*S* = 3,*V* = 80	81.8083 ± 1.1091	76.3833 ± 2.5471	87.2333 ± 0.8791	0.9093 ± 0.0045
*S* = 4,*V* = 80	75.9111 ± 1.4438	65.4444 ± 2.7542	86.3778 ± 1.2067	0.8781 ± 0.0066

From Table 3, we can find that the classification rsults is best when the window width is set to 60 and the step size is set to 2. When the step size is set to 1∼3, the classification performance is higher when the window width is 70. As the step size increases, the classification performance shows a downward trend. This is consistent with the conclusion of Jiao et al. (2019). The season is that a larger step size tends to ignore the part of the dynamic information of the functional connection between regions that changes over time, and the classification performance gradually decreases.

### Discriminative Brain Regions and Functional Connections

**FIGURE 4:**
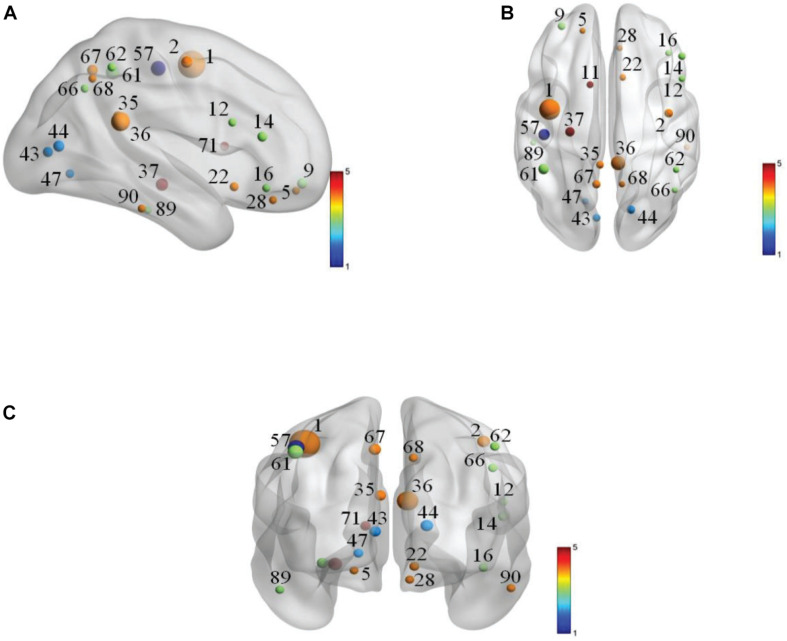
Visualization of discriminative brain regions. **(A)** Cornal plane. **(B)** Axis plane. **(C)** Sagittal plane.

The selected features in each 10-fold cross-validation are different, namely, the selected weighted-graph local clustering coefficients are different, so we count the features that have been selected more times in ten times of 10-fold-cross-validation. These features correspond to 24 brain regions, known as discriminative brain regions. Table 4 shows the discriminative brain regions, which are visualized by using BrainNet Viewer toolbox^4^ (Xia et al., 2013). Figure 4 shows the visualization results of these discriminative brain regions on the Jet template, where each node corresponds to a brain region.

**TABLE 4:** Discriminative brain regions.

		**MNI coordinates**			
	**Abbreviations**				
**ID**	**(L: left, R: right)**	**X (mm)**	**Y (mm)**	**Z (mm)**	**References**
1	PreCG.L	–38.65	–5.68	50.94	
2	PreCG.R	41.37	–8.21	52.09	Zhang et al., 2016a
5	ORBsup.L	–16.56	47.32	–13.31	Xu et al., 2016
9	ORBmid.L	–30.65	50.43	–9.62	Zhang et al., 2018
12	IFGoperc.R	50.20	14.98	21.41	Chen et al., 2016
14	IFGtriang.R	50.33	30.16	14.17	Salvatore et al., 2015
16	ORBinf.R	41.22	32.23	–11.91	Salvatore et al., 2015
22	OLF.R	10.43	15.91	–11.26	Sun et al., 2012
28	REC.R	8.35	35.64	–18.04	
35	PCG.L	–4.85	–42.92	24.67	Zhang et al., 2018
36	PCG.R	7.44	–41.81	21.87	Wee et al., 2012
37	HIP.L	–25.03	–20.74	–10.13	Salvatore et al., 2015
43	CAL.L	–7.14	–78.67	6.44	Xu et al., 2016
44	CAL.R	15.99	–73.15	9.40	
47	LING.L	–14.62	–67.56	–4.63	
57	PoCG.L	–31.16	–40.30	–20.23	Xu et al., 2016
61	IPL.L	–42.80	–45.82	46.74	
62	IPL.R	46.46	–46.29	49.54	Salvatore et al., 2015
66	ANG.R	45.51	–59.98	38.63	Xu et al., 2016
67	PCUN.L	–7.24	–56.07	48.01	
68	PCUN.R	9.98	–56.05	43.77	
71	CAU.L	–11.46	11.00	9.24	Salvatore et al., 2015
89	ITG.L	–49.77	–28.05	–23.17	Zhang et al., 2018
90	ITG.R	53.69	–31.07	–22.32	

In Table 4, we find that some regions corresponding to features in the default mode network (DMN) are selected, such as left posterior cingulate gyrus (PCG.L), right posterior cingulate gyrus (PCG.R), left hippocampus (HIP.L), left inferior parietal, supramarginal and angular gyri (IPL.L), right inferior parietal, supramarginal and angular gyri (IPL.R), right angular gyrus (ANG.R), left precuneus (PCUN.L), right precuneus (PCUN.R), left inferior temporal gyrus (ITG.L), and right inferior temporal gyrus (ITG.R), etc. Most of the selected brain regions have been widely considered to be related to AD. Taking left hippocampus (HIP.L) as an example, the hippocampus structure is considered to play an important role in memory, spatial navigation, and attention control, and the hippocampus structure is also the first part to lose the memory and disorientation damage. It was found that the hippocampus structure of AD patients has been severely damaged. The left hippocampus (HIP.L) is selected, indicating that eMCI patients already have a chance to transition to AD, and it also shows that DMN plays an important role in cognitive function (Jiao et al., 2017a,b, c). In addition, some brain regions belong to the frontal lobe are extracted, such as left superior frontal gyrus, orbital part (ORBsup.L), left middle frontal gyrus, orbital part (ORBmid.L), right inferior frontal gyrus, opercular part (IFGoperc.R), right inferior frontal gyrus, triangular part (IFGtriang.R), right inferior frontal gyrus, orbital part (ORBinf.R), right olfactory cortex (OLF.R), and right gyrus rectus (REC.R), etc. It demonstrates that the language and mental activities of eMCI patients have changed a lot compared with normal people (Wee et al., 2011).

**FIGURE 5:**
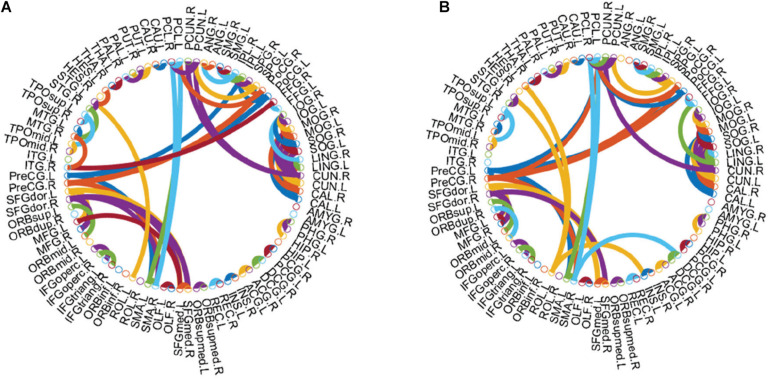
Top 100 functional connections with the highest correlation coefficients in AFN. **(A)** Normal subjects. **(B)** eMCI subjects.

**FIGURE 6:**
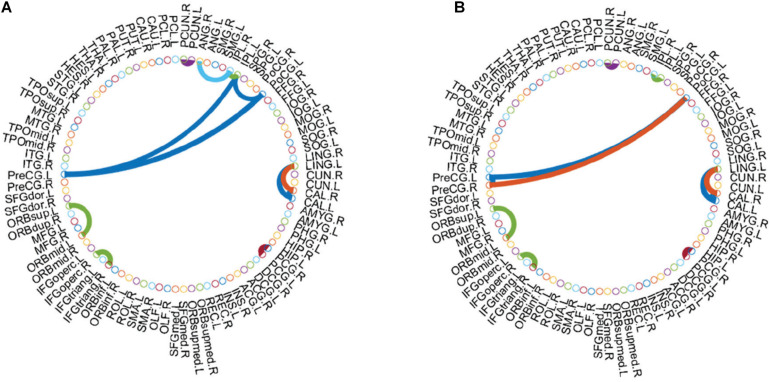
Functional connections between discriminative regions in AFN. **(A)** Normal subjects. **(B)** eMCI subjects.

In order to explore the relationships between discriminative brain regions, we first sum all WRTDFNs of each type of subjects and then average them to obtain their average functional network (AFN), respectively. As there are many functional connections in AFN, the effect is not obvious after all of them are visualized, and a specific number of functional connections are selected for visualization. Related studies have shown that it is more obvious to select a specific number of functional connections with the highest correlation coefficient as significant functional connections (Ou et al., 2015; Jiao et al., 2020). Accordingly, we select the top 100 functional connections with the highest correlation coefficients as the significant functional connections for visualization. On this basis, we also find the functional connections between discriminative brain regions for further analyzing the physiological significance of DFN. Figures 5A,B, respectively, show the top 100 functional connections with the highest correlation coefficients in the AFNs of the two types of subjects. Figures 5B, 6A, respectively, show the functional connections between discriminative brain regions of the two types of subjects, where the width of the arc represents the connection strength between two brain regions.

As shown in Figure 5, there are some Hub nodes in the AFN of normal subjects and eMCI subjects, and the Hub nodes are only a small proportion of the whole brain regions, illustrating the scale-free property of the AFN. The Hub nodes of the normal subjects are left precental gyrus (PreCG.L), right precental gyrus (PreCG.R), left superior frontal gyrus, dorsolateral (SFGdor.L), right superior frontal gyrus, dorsolateral (SFGdor.R), left postcentral gyrus (PoCG.L), left calcarine fissure and surrounding cortex (CAL.L), right calcarine fissure and surrounding cortex (CAL.R), right cuneus (CUN.R), and left precuneus (PCUN.L). The Hub nodes of the eMCI subjects are left precental gyrus (PreCG.L), right precental gyrus (PreCG.R), left superior frontal gyrus, dorsolateral (SFGdor.L), right superior frontal gyrus, dorsolateral (SFGdor.R), right postcentral gyrus (PoCG.R), and right rolandic operculum (ROL.R). These nodes are mainly concentrated in the prefrontal lobe, which shows that these parts of normal subjects and eMCI subjects are relatively active, involving some complex cognition, such as memory, judgment, analysis, thinking, and manipulation. Comparatively, functional connections have changed in the prefrontal lobe of eMCI subjects. While in Figure 6, the same functional connectivity between the discriminative regions in two types of subjects are PCUN.L-PUCN.R, IPL.L-IPL.R, PreCG.L-PoCG.L, ORBsup.L-ORBmid.L, IFGoperc.R-IFGtriang.R, PCG.L-PCG.R, LING.L-CAL.L, and LING.L-CAL.R, among which IFGoperc.R-IFGtriang.R and ORBsup.L-ORBmid.L are consistent with previous studies (Jiao et al., 2019; Li et al., 2020). Moreover, compared with the normal subjects, eMCI subjects have different functional connections between the discriminative brain regions, such as PreCG.R-PoCG.L and PreCG.L-PreCG.R, which may be of great significance to the diagnosis of eMCI.

## Discussion

We propose a method for constructing DFNs based on WR and TLA and apply it to eMCI classification. First, we formulate the DFN construction method based on PC into an optimization model, and add a WR term to obtain a WRDFN, and then concatenate the WRDFNs of all subjects into a tensor for TLA processing. The DFN of each subject in the tensor after TLA is its WRTDFN. Finally, the obtained WRTDFNs are used to classify eMCI subjects and normal subjects, and the classification performance is better than the comparable methods.

By the results above, we find that PC method with a regularization term has generally better classification performance for eMCI than the SR method with a regularization term. The reason is mentioned in the research by Li et al. (2017) and Qiao et al. (2016). Since SR involves the inverse operation of the covariance matrix, there may be an ill-posed problem (Michel and Telschow, 2016). It means that the singularity of the covariance matrix makes it impossible to directly inverse the covariance matrix. PC does not involve inverse operations, thus adding a regularization term can better construct DFNs. In addition, WRTLA achieves better classification performance than PC_scale–free_, although both of them all involve WR. One of the important reasons is that PC_scale–free_ has the function of modeling Hub nodes in brain functional networks, which can cover functional connections closely related to neural disorders. We develop WRTLA to construct DFNs through a unified learning framework that integrates functional connection strength, sparsity, and group constraints, and the obtained DFNs are also more biologically meaningful.

However, WRTLA also has some limitations, which need to be improved in our future work. First, the topological properties of brain functional networks are far more than sparsity, low-rank property, and scale-free property. How to use other topology properties of complex networks as prior information to construct the optimal brain functional network is a worthy problem. In addition, we focus on construction of DFN, and we apply the *t*-test for feature selection. The improvement strategies are as follows: simply improving the feature selection and combining the test dataset on the training dataset to select features iteratively, and gradually select the features that improve the classification performance.

In conclusion, WRTLA utilizes some functional connections between discriminative regions to construct DFNs. The innovation of WRTLA is that, it solved the problems that most methods cannot properly reflect the similarities and differences of functional connections among different people in constructing brain functional networks. We apply WRTDFNs to classify eMCI subjects and normal subjects, and the experimental results show that the classification performance of this method is better than other methods. Our work can effectively improve the classification performance of DFN constructed by existing methods for eMCI, and has certain reference value for the early diagnosis of AD. Additionally, the above work will provide some enlightenment for us to carry out other auxiliary diagnosis of cognitive disorders in the future.

## Data Availability Statement

All datasets generated for this study are included in the article/supplementary material.

## Ethics Statement

The studies involving human participants were reviewed and approved by the Biomedical Ethics Committee of the Changzhou University. The patients/participants provided their written informed consent to participate in this study.

## Author Contributions

ZJ: methodology and writing. YJ: writing and visualization. JZ: data processing. HS: data processing and verification. CW: methodology and modification. All authors contributed to the article and approved the submitted version.

## Conflict of Interest

The authors declare that the research was conducted in the absence of any commercial or financial relationships that could be construed as a potential conflict of interest.

## Footnotes

1 http://adni.loni.ucla.edu

2 http://www.fil.ion.ucl.ac.uk

3 http://rfmri.org/dparsf

4 https://www.nitrc.org/projects/bnv/
